# Computer Vision for Brain Disorders Based Primarily on Ocular Responses

**DOI:** 10.3389/fneur.2021.584270

**Published:** 2021-04-21

**Authors:** Xiaotao Li, Fangfang Fan, Xuejing Chen, Juan Li, Li Ning, Kangguang Lin, Zan Chen, Zhenyun Qin, Albert S. Yeung, Xiaojian Li, Liping Wang, Kwok-Fai So

**Affiliations:** ^1^Brain Cognition and Brain Disease Institute, Shenzhen Institutes of Advanced Technology, Chinese Academy of Sciences, Shenzhen, China; ^2^Shenzhen-Hong Kong Institute of Brain Science-Shenzhen Fundamental Research Institutions, Shenzhen, China; ^3^Department of Brain and Cognitive Sciences, Massachusetts Institute of Technology, Cambridge, MA, United States; ^4^BIAI INC., Chelmsford, MA, United States; ^5^BIAI Intelligence Biotech LLC, Shenzhen, China; ^6^Department of Neurology, Harvard Medical School, Harvard University, Boston, MA, United States; ^7^Retina Division, Department of Ophthalmology, Boston University Eye Associates, Boston University, Boston, MA, United States; ^8^Center for High Performance Computing, Shenzhen Institutes of Advanced Technology, Chinese Academy of Sciences, Shenzhen, China; ^9^Department of Affective Disorders and Academician Workstation of Mood and Brain Sciences, The Affiliated Brain Hospital of Guangzhou Medical University (Guangzhou Huiai Hospital), Guangzhou, China; ^10^Guangdong-Hong Kong-Macau Institute of Central Nervous System (CNS) Regeneration, Jinan University, Guangzhou, China; ^11^Key Laboratory for Nonlinear Mathematical Models and Methods, School of Mathematical Science, Fudan University, Shanghai, China; ^12^Depression Clinical and Research Program, Department of Psychiatry, Massachusetts General Hospital, Boston, MA, United States; ^13^The State Key Laboratory of Brain and Cognitive Sciences, Department of Ophthalmology, University of Hong Kong, Pok Fu Lam, Hong Kong

**Keywords:** ocular assessment, retina, computer vision, cognitive neuroscience, brain disorders, eye-brain engineering

## Abstract

Real-time ocular responses are tightly associated with emotional and cognitive processing within the central nervous system. Patterns seen in saccades, pupillary responses, and spontaneous blinking, as well as retinal microvasculature and morphology visualized via office-based ophthalmic imaging, are potential biomarkers for the screening and evaluation of cognitive and psychiatric disorders. In this review, we outline multiple techniques in which ocular assessments may serve as a non-invasive approach for the early detections of various brain disorders, such as autism spectrum disorder (ASD), Alzheimer's disease (AD), schizophrenia (SZ), and major depressive disorder (MDD). In addition, rapid advances in artificial intelligence (AI) present a growing opportunity to use machine learning-based AI, especially computer vision (CV) with deep-learning neural networks, to shed new light on the field of cognitive neuroscience, which is most likely to lead to novel evaluations and interventions for brain disorders. Hence, we highlight the potential of using AI to evaluate brain disorders based primarily on ocular features.

## Introduction

The neurosensory retinas play a critical role in the functioning of our central nervous system (CNS), the latter of which processes our sensory input, motor output, emotion, cognition, and even consciousness (1). Multiple studies have shown that ocular evaluations can be used to assess CNS disorders (2). Many neurological and psychiatric disorders—such as glaucoma, stroke, Parkinson's disease (PD), autism spectrum disorder (ASD), Alzheimer's disease (AD), major depressive disorder (MDD), and schizophrenia (SZ)—lead to considerable personal suffering, financial costs, and social burden (3). Distinct ocular findings have exhibited the possibility of ocular assessments as early biomarkers for these disorders (2, 4). Since brain disorders represent one of the most challenging issues to modern humans, indeed, novel approaches are needed to advance psychiatric medicine, especially in terms of objective cognitive measurements (5) and real-time interventions for cognitive problems (6).

**Figure 1:**
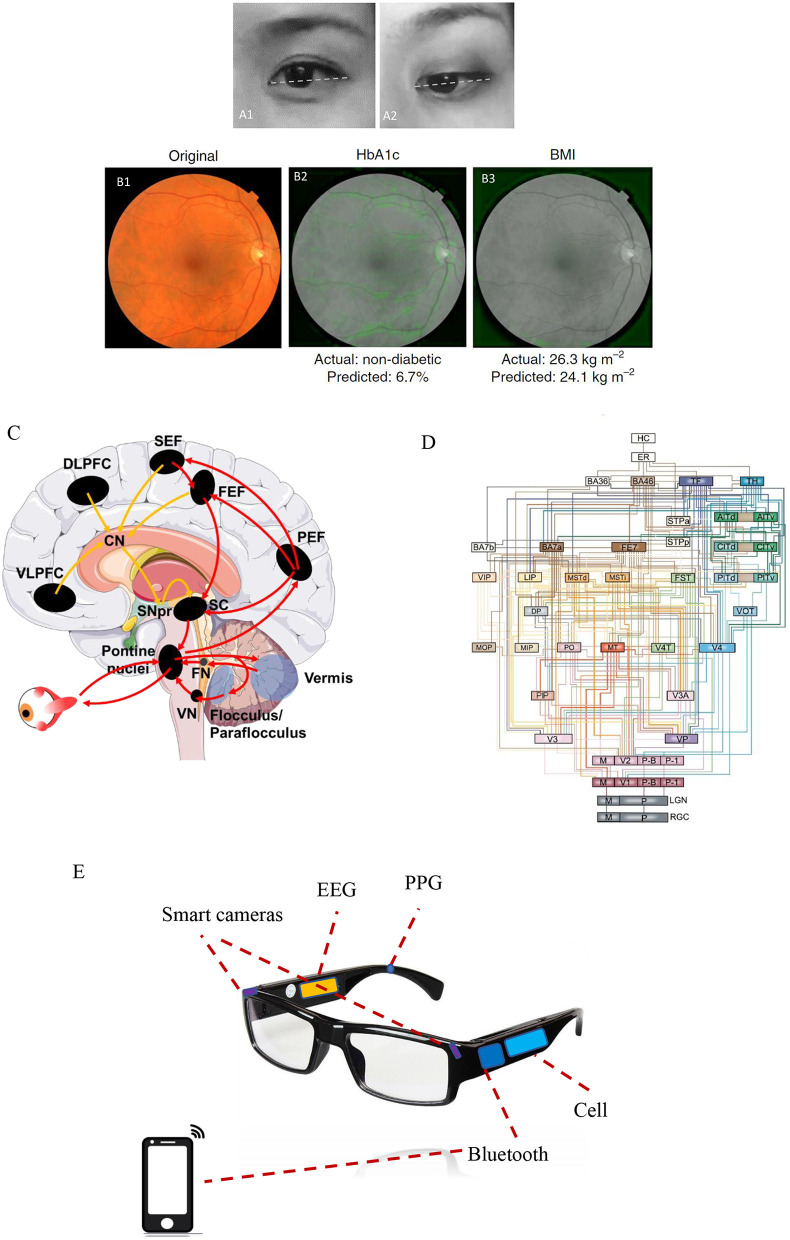
The eye as a window to uncover a healthy level of the brain. **(A1)** A restful and calm eye (positive state) is shown compared with **(A2)** a stressful and anxious eye (negative state). Note that a positive state is more frequently associated with an upside view of the eyes, whereas a negative state exhibits a more downside view of eyes. The eye images shown here are presented following permission from the corresponding subjects. **(B1)** Example of a retinal fundus image in color, whereas **(B2, B3)** show the same retinal image but in black and white. Machine learning predictions of diabetes and body mass index (BMI) states mainly rely on the features of the vasculature and optic disc, as indicated by the soft attention heat map with green color in those images. The images in **(B1–B3)** were adapted from Poplin et al. (8) (with permission). **(C)** Complex neural networks spanning the cortical, subcortical, and cerebellar areas are involved in voluntary saccadic eye movements for attentional control. The image was modified from that of Johnson et al. (11). Red arrows indicate the direct pathway (PEF, the parietal eye fields; FEF, frontal eye field; SEF, supplementary eye field) to the superior colliculus (SC) and brainstem premotor regions, while yellow arrows indicate the indirect pathway to the SC and brainstem premotor regions via the basal ganglia (striatum, subthalamic nucleus, globus pallidus, and substantia nigra pars reticularis). **(D)** An architectural model of the hierarchy of visual cortical circuitry, modified from Felleman and Van (12). There is a feedforward ascending pathway of the vision system from the retinas to the cortex, as well as a feedback descending pathway from the cortex to multiple downstream areas. **(E)** A potential application of eye–brain engineering developed to compute human brain states mainly based on smart cameras to detect ocular responses, combined with other biological signals including electroencephalography (EEG) and photoplethysmography (PPG).

Recently, the Google/DeepMind was able to detect retinal diseases and cardiovascular risk factors using artificial intelligence (AI) algorithms on retinal images (7, 8). AI studies have already shown that AI-based detection to various diseases is possible and the potential of AI to impact the next generation of medical care as well (9). Given the increasing data connecting ocular parameters with brain disease states, it is possible that applying AI algorithms on ocular patterns would be helpful for the detection and evaluation of these diseases, especially with the rapid advancement of computer vision (CV) and deep-learning algorithms [(9, 10); Figures 1A,B]. In this present review, we discuss and highlight a novel approach of using CV with advanced AI to evaluate human brain disorders based primarily on ocular responses.

## Eye–Brain Connection

The eyes and the brain are intimately connected. Approximately 80% of the sensory input to the human brain initiates from the vision system, which begins at the retinas (1, 2). The axons of retinal ganglion cells (RGCs) send visual information collected on the neurosensory retinas to the CNS. There are at least 20 nuclei that receive projections from the retinas into the mammalian brain (13, 14); for example, the lateral geniculate nucleus serves as a thalamic visual relay to primary visual cortex, the superior colliculus is responsible for visuomotor processing, and the hypothalamic suprachiasmatic nucleus is involved in non-visual hormonal photoentrainment (15).

The eyes are usually linked to facial expressions, such as eye widening can be a sign of fear, whereas eye narrowing can be a sign of disgust (16). Lee et al. demonstrated that eye widening enhances one's visual field, thereby improving stimulus detection, while eye narrowing increases visual acuity, which improves objective discrimination (17, 18). Moreover, visual attention, pupillary responses, and spontaneous blinking are regarded as non-invasive and complementary measures of cognition (19). Vision-based attentional control includes the planning and timing of precise eye movements, which has been shown to be controlled by neural networks spanning cortical, subcortical, and cerebellar areas that have been extensively investigated in both humans and non-human primates [(20–22); Figure 1C]. Pupillary responses are primarily modulated by norepinephrine in the locus coeruleus, which controls physiological arousal and attention (23–25), whereas spontaneous eye-blink rates are tightly correlated with CNS dopaminergic levels and are associated with processes underlying learning and goal-directed behavior [(26, 27); Figure 1D]. Below, we outline in detail the connections between ocular assessments and some brain disorders including autism spectrum disorder (ASD), Alzheimer's disease (AD), schizophrenia (SZ), and major depressive disorder (MDD).

## Autism Spectrum Disorder: Without Normal Eye Contact

A lack of normal eye contact during social interaction is one of the main clinical features of ASD (28). Screening for ocular fixation at 2–6 months old can provide early detection and even interventions for children with ASD (29). Full-field electroretinogram (ERG), which measures specific cellular functions within the retinas, further exhibits decreases in rod b-wave amplitude in ASD individuals (30). Different types of oculomotor dysfunction—such as saccade dysmetria (over- or undershooting of visual targets), loss of saccadic inhibition, and fixation impairment—have all been documented in patients with ASD (11). Saccade dysmetria may be caused by the dysfunction of neural networks connecting the cerebellar vermal–fastigial circuitry to the brainstem premotor nuclei that regulate oculomotor movements [(28, 29); Figure 1C]. A diminished ability to inhibit reflexive saccades during anti-saccade tasks is thought to be associated with the characteristic repetitive behaviors seen in patients with ASD (31, 32). In addition, fixation is often significantly impaired in ASD patients, which is more likely secondary to a reduced top–down modulation of sensorimotor processing (33, 34).

## Alzheimer'S Disease: Dementia Feature in the Eyes

Ocular assessments of AD patients have demonstrated saccadic dysfunctions indicative of poor visual attention. In particular, AD patients have difficulty focusing on fixed objects (35). Prettyman et al. showed early in 1997 that there was a 75% greater latency in pupillary constriction in AD patients compared with that in age-matched controls (36). Additionally, AD patients have markedly decreased visual contrast sensitivity, which is evident even at the early stage of AD (37). Patients with AD also have altered retinal microvasculature, such as sparser and more tortuous retinal vessels and narrower retinal venules (38) and decreased retinal blood flow/blood-column diameter as indicated by laser Doppler flowmetry (39). Studies using optical coherence tomography (OCT) have indicated a gradual decrease in retinal nerve fiber layer thickness (RNFL), most prominently in the superior quadrants, when comparing patients with no AD to mild AD to severe AD (40, 41). AD patients exhibit a number of specific ocular findings; these ocular findings provide an opportunity for their collective use as biomarkers for machine learning algorithms to test AD (42).

## Schizophrenia: To See or Not to See

Visual processing impairment including visual hallucination, distortion of shapes, or light intensity, is commonly observed in patients with SZ (43). Abnormal retinal findings like dilated retinal venules, RNFL thinning, and ERG abnormalities were present in SZ patients (44, 45). A twin study showed a positive correlation between wider retinal venules and more severe psychotic symptoms (46), suggesting the possible use of retinal venule diameter as a biomarker for SZ. In addition, RNFL thinning, which corresponds to the loss of RGCs axons, is seen in SZ patients (47, 48) and also in patients with PD (49) and AD (50). ERG abnormalities in SZ indicate reduced functionalities of rod and cone photoreceptors, bipolar cells, and RGCs, all of which can reflect the deregulation of neurotransmitters such as dopamine (51, 52). Furthermore, a portable handheld ERG device was made and can be used in psychiatry clinics for screening and evaluation of SZ (53).

## Major Depressive Disorder: A Gray World of Eyes

Reduced contrast sensitivity is frequently seen in individuals with MDD, both medicated and unmedicated (54). There is even a strong correlation between contrast gain and depression severity, as indicated by pattern electroretinogram (PERG) (54, 55). When compared with healthy controls, patients with MDD often have higher error rates and increased reaction times in performing anti-saccade tasks (56, 57). Furthermore, patients with melancholic depression, when compared with saccade parameters in healthy controls and non-melancholic depressed patients, exhibit longer latencies, reduced peak velocities, and greater hypometricity during saccadic eye movement tasks (58). Patients with seasonal affective disorder (SAD, namely, winter depression) have a significantly reduced post-illumination pupillary response (PIPR) as demonstrated by infrared pupillometry (59, 60). These features are most likely to associate with dysfunction of melanopsin-expressing intrinsically photosensitive RGCs (ipRGCs), since ipRGCs often contribute to pupillary response function, particularly during sustained-state pupillary constriction (61, 62). However, the change in PIPR in response to blue light stimuli only happened in SAD patients carrying the OPN4 I394T genotype (59).

As previously outlined, different brain disorders usually have ocular manifestations. However, those ocular features may be either shared among multiple diseases or specific to a singular disease, as shown in detail in Table 1. Vast psychological and economic burdens caused by brain disorders call for more precise analyses of those disorders. It is a good choice for analysis in advance to combine with machine learning particularly deep-learning algorithms.

**Table 1:** Multiple changes in ocular parameters via ophthalmological assessments are associated with neurological disorders.

	**Saccades**	**Pupillary/blinking response**	**RNFL**	**Microvasculature**	**ERG**
Autism	Decrease eye fixation at 2–6 months old (29); saccade dysmetria (11); impaired tracking of moving targets (33)	A longer latency of the blink reflex in high-functioning autism (63)	**–**	**–**	Decreased rod b-wave amplitude in flash ERG (30)
Alzheimer's disease	Poor eye fixation (35)	Delayed pupillary constriction (36, 60)	Reduced RNFL thickness especially in the superior quadrant (40, 41)	Narrower retinal venules and sparser and more tortuous retinal vessels (38)	Markedly decreased contrast sensitivity (37)
Schizophrenia	Performed worse in predictive, reflexive, and antisaccade tasks (64)	Blink rates are frequently elevated (65)	Thinning of RNFL (47, 48)	Widened retinal venules (46)	Abnormal ERG amplitudes including rods, cones, bipolar cells, and RGCs (53)
Major depression	Elevated error rates and increased reaction times (56, 57)	Reduced PIPR and a lower PIPR percent change in response to blue light in patients with SAD (59)	**–**	**–**	Significantly reduced contrast sensitivity using PERG (54, 55)

*Some similar features among these diseases further indicate a requirement of more precise analyses via machine learning and deep learning. ERG, electroretinogram; PERG, pattern electroretinogram; PIPR, post-illumination pupillary response; RNFL, retinal nerve fiber layer thickness; SAD, seasonal affective disorder*.

## Computer Vision: With Advanced Artificial Intelligence

CV, as one of the most powerful tools to push AI applications into healthcare areas, exhibits a high capability of auto-screening diseases, such as skin cancer (66) and diabetic retinopathy (10, 67). Following a rapid development of deep learning-based AI, CV now has an impressive resolution that is close to that of human vision and maybe beyond sometime (9, 68). Hence, CV is likely to drive AI to provide novel tools for brain disorders, as machines have now been able to be trained to read human emotional and cognitive states, especially in terms of automated detection of facial expressions like fear and fatigue (18, 69). Individuals with brain disorders may benefit from CV-based AI applications in neurological healthcare, particularly to aid in patient self-monitoring of symptoms and in conducting real-time interventions for recovery of social and psychological abilities (70). Furthermore, CV-based AI recognition of human emotional and cognitive states will be more precisely achieved with automated detection and analyses of ocular responses.

Nevertheless, currently available CV datasets on emotional recognition—such as JAFFE, FERA, and CK^+^–have usually been based on thousands of facial images captured in the laboratory, but often neglected human eye movement and other primary ocular parameters, which usually contain abundant information on human affective states (16). Since Google AI applications focused on retinal and eye features have already demonstrated that this approach non-invasively and conveniently yields much information of physical health (7, 10), such eye-focused CV-based AI should be explored further for clinical neurosciences in the future. Hence, the eye as a window into the brain can be used to obtain health information, not only for eye diseases but also for determining cardiovascular risk factors (8) and even for brain disorders (Table 1). In order to achieve reliable detection of human emotional and cognitive states for brain disorders, a more quantitative representation of emotional recognition via AI algorithms is necessary, which will require more informative databases with dynamic features including eye movements captured with the natural responses, and furthermore, investigations to the neural mechanism involved action units of facial expression as well (71).

## Discussion

Medical issues engaged in challenging human diseases are often tightly associated with big data, and AI algorithms have been demonstrated to leverage such big data to aid in solving these issues (9). AI applications in medicine are only at an early stage; however, AI-based automated diagnoses have already contributed to the identification of several types of cancers and retinal diseases (10, 66, 67). Some case reports on AI practices engaged in medical diagnoses have involved image recognition via supervised learning using deep neural networks, which has helped in effectively interpreting cancer slides (72), retinal images (73), and brain scans as well (74); nevertheless, most of these applications have only been completed at the preliminary stage.

AI performance has been frequently leveraged in ophthalmology since retinal images are relatively easy to obtain using fundus imaging or OCT without any invasion. Additionally, diagnostic standards of eye diseases have become more well-defined. Many eye diseases—including diabetic retinopathy (75), age-related macular degeneration (76), and congenital cataracts (77)—have already been assessed via deep-learning neural networks, and many applications have exhibited remarkable accuracies comparable with those of eye specialists (9). Since the neurosensory retinas as a key component of the vision system is a direct embryological extension of the CNS (1), the utility of AI algorithms to detect abnormal ocular responses associated with brain disorders is reasonable to test for its feasibility and efficacy (2, 78). CV-based AI algorithms for automated detection and analyses of ocular responses is more likely to represent promising tools for non-invasively detecting differences in ocular responses (16), particularly those associated with different types of brain disorders (78). Brain disorders tend to be more complicated than other medical issues, and often lead to a greater burden to human society, as indicated by at least 350 million people currently suffering from major depression in the world (79). There is a huge potential for AI to lend its power to patients with affective disorders and the caregivers helping these patients.

Undoubtedly, AI has begun to shed new light on brain disorders. Cognoa applied clinical data from thousands of children at risk for ASD to train and develop an AI platform, which may provide earlier diagnostics and personalized therapeutics for autistic children (80) and was approved by the FDA in 2018. In terms of autoscreening depression, Alhanai et al. used audio and text features to train a neural network with long short-term memory, which was found to be comparable with traditional evaluations via depression questionnaires (81). An eye tracking-based assessment has been already developed with video movies shown at the monitor, in order to evaluate cognitive impairments such as ASD and AD (82, 83). Haque et al. trained their AI using spoken language and 3D facial expressions commonly available in smartphones to measure depression severity (84). A project in our team is currently running and is aimed at developing an AI platform that utilizes ocular data to train a model to detect brain states under natural conditions. This AI platform is designed mainly based on real-time ocular responses and is likely to determine brain emotional and cognitive states of individuals with brain disorders. This core function will be accessed through a wearable smart glasses and an ordinary smartphone (in Figure 1E, the related patent was in progress).

Nevertheless, some issues regarding AI implementation in healthcare require consideration. The first issue is how to effectively collect big data with a high quality of valid features for AI algorithms. In terms of AI recognition of facial expression, high-resolution imaging is often required, especially for the potential application of brain disorders. Multiple ocular data should not be neglected for emotional recognition since eye expression plays a key role in social communication (16). Parameter patterns such as saccades, pupillary response, and blinking rate (Table 1) contain detailed and fruitful information on human affective states (19). Micro-expression detection is also sometimes required for the ground truth of facial expression (85). Some key features are unable to be shown by one single image and, instead, require dynamic videos with high frame rates. In addition, high-resolution imaging is potentially beneficial for obtaining more healthcare data via photoplethysmography (PPG) (86), such as heart rate variability (87). Another key issue is in terms of privacy protection when facial data are obtained to develop AI algorithms (88). Participants need to be provided informed consent regarding data collection. Investigators need to advise participants of their rights, summarize what is expected for participation during the study, and then keep the data safety continuously after the study (88). In our pilot design, all facial or ocular data will be obtained and stored by participants themselves through their smartphones, and they have the right to decide if data are shared without identifying their personal information. The third issue is some portable or wearable devices are required to develop for data collection of brain healthcare. It is better for early detection of symptoms involved in brain disorders (70), rather than diagnosing symptoms at the later and severe stages using functional MRI for evaluating depression (89) and computed tomography (CT) for screening head trauma (90). The wearable smart glasses that we designed (Figure 1E) can collect many different parameters involving ocular responses like saccades, pupillary response, and blinking parameters. Also, other biological data will be considered as well, such as electroencephalography (EEG) and PPG (Figure 1E). It will be defined as an integrated eye–brain engineering tool for human state recognition that enables powerful detection and evaluation of brain states in real time with machine learning. It is more likely to benefit some patients with brain disorders, even including stroke and bipolar disorders.

As AI is still at the early stage of being integrated into mental healthcare, integration of human biological intelligence (BI) and machine learning-based AI will need to be further promoted. AI alone is known to be insufficient for detecting brain disorders since machine learning is entirely dependent on the availability of collected data. The quality of collected data is vital, which will require the use of more knowledge from BI. Current AI representation of human facial expressions is often only achieved at a qualitative level that has been categorized into seven basic expressions plus some composite expressions, with an accuracy ratio of <70% (91, 92). Therefore, it requires the improvement to a quantitative level via more BI, especially with cognitive neuroscience and neuro-ophthalmology. Then we can perhaps learn more clearly about the threshold values of brain disorders distinguished from normal brain functions and learn more about specific features of different brain disorders. More interestingly, vision intervention with some ocular responses also directly exhibited the benefits to rescue brain disorders, for example, blue-enriched light therapy to major depression (14, 93) and 40-Hz light flicker to attenuate AD-associated pathology (94, 95). This type of vision-based therapy may be much beneficial for conducting non-invasive and timely prevention and even treatment for brain disorders in the near future. To this aim, machine learning-based AI, especially deep-learning neural networks, will likely be instrumental in further advances in clinical neuroscience (96, 97).

## Conclusion and Perspective

In general, brain disorders can be assessed by ocular detection, while that certainly needs to consider the exclusion of the eye disease situation and that well-trained AI will offer its support again (7). After all, advanced AI will help those patients with brain disorders directly and currently, differing from gene therapy, which often plans to benefit for the next generation of those patients. It can be achieved mainly through autodetection with AI algorithms, self-evaluation with wearable sensors, and timely intervention with brain–computer interface as well. May the force of AI be with patients of brain disorders particularly using an approach with ocular representation in the real time.

## Data Availability Statement

The raw data supporting the conclusions of this article will be made available by the authors, without undue reservation.

## Ethics Statement

The studies involving human participants were reviewed and approved by Institute Review Board in Shenzhen Institutes of Advanced Technology, Chinese Academy of Sciences, China. The patients/participants provided their written informed consent to participate in this study. Written informed consent was obtained from the individual(s) for the publication of any potentially identifiable images or data included in this article.

## Author Contributions

XiaotL wrote this works. FF drawed the Figures 1C,D. XC and K-FS revised this manuscript. JL and ZC collected some data. LN and ZQ worked for machine learning and computer vision. KL and AY reviewed the part of brain disorders. XiaojL and ZC designed the engineering of smart glasses. KF-S and LW supported and guided this project. All authors contributed to the article and approved the submitted version.

## Conflict of Interest

XL and JL were both core members at the start-up company of BIAI INC, USA/BIAI LLC., China. The remaining authors declare that the research was conducted in the absence of any commercial or financial relationships that could be construed as a potential conflict of interest.
